# A Large-Scale Survey of the Bacterial Communities in Lakes of Western Mongolia with Varying Salinity Regimes

**DOI:** 10.3390/microorganisms8111729

**Published:** 2020-11-04

**Authors:** Kshitij Tandon, Bayanmunkh Baatar, Pei-Wen Chiang, Narangarvuu Dashdondog, Bolormaa Oyuntsetseg, Sen-Lin Tang

**Affiliations:** 1 Biodiversity Research Center, Academia Sinica, Taipei 115, Taiwan; kshitijtandon@gate.sinica.edu.tw (K.T.); b.bayanmunkh@mnun.edu.mn (B.B.); momo12390@gmail.com (P.-W.C.); 2 Bioinformatics Program, Institute of Information Science, Taiwan International Graduate Program, Academia Sinica, Taipei 115, Taiwan; 3 Institute of Molecular and Cellular Biology, National Tsing Hua University, Hsinchu 300, Taiwan; 4 School of Arts and Sciences, National University of Mongolia, Ulaanbaatar 14201, Mongolia; garvuu@num.edu.mn

**Keywords:** bacterial community, saline lakes, western Mongolia, *Cyanobium*-PCC6307, *Halomonas*, *Spiribacter*, local and regional factors

## Abstract

In recent years, climate change coupled with anthropogenic activities has led to monumental changes in saline lakes which are rapidly drying up across the globe and particularly in Central Asia. The landlocked country of Mongolia is rich in lakes which have remained primarily undisturbed by human impact, and many of these lakes have varying salinity regimes and are located across various geographical landscapes. In this study, we sampled 18 lakes with varying salinity regimes (hyperhaline, mesohaline, oligohaline, and polyhaline) covering 7000 km of western Mongolia and its various geographical landscapes (Gobi Desert, forests, and steppe). We identified that the bacterial communities that dominate these lakes are significantly influenced by salinity (*p* < 0.001) and geographical landscape (*p* < 0.001). Further, only five zOTUs were shared in all the lakes across the salinity regimes, providing evidence that both local and regional factors govern the community assembly and composition. Furthermore, the bacterial communities of hyperhaline lakes were significantly positively correlated with salinity (ANOVA, *p* < 0.001) and arsenic concentrations (ANOVA, *p* < 0.001), whereas bacterial communities of mesohaline and polyhaline lakes situated in forest and steppe landscapes were positively correlated with temperature (ANOVA, *p* < 0.001) and altitude (ANOVA, *p* < 0.001), respectively. Functional predictions based on the 16S rRNA gene indicated enrichment of KEGG Ontology terms related to transporters for osmoprotection and -regulation. Overall, our study provides a comprehensive view of the bacterial diversity and community composition present in these lakes, which might be lost in the future.

## 1. Introduction

Lakes form a major proportion of aquatic ecosystems on a global scale. In particular, saline lakes account for ~44% and ~23% of the total volume and area of global lakes, respectively. These saline lakes are predominantly located in arid, endorheic basins [1]. Furthermore, these lakes are sentinels for anthropogenic activities and associated global warming [2]. In recent years, saline lakes around the world have seen a significant decline, including those present in remote areas of the Mongolian Plateau [1,3]. The effect of global warming on lakes is complemented by the removal of freshwater from lake drainage areas for agricultural, industrial, and municipal applications [4], leading to increased evaporation rates and over-salting [5]. While these activities change the biogeochemical properties of lakes, they also affect the lakes’ microbial diversities. Since microbes contribute significantly to the biogeochemical cycles at a global scale [6], it is important to study and delineate the microbial diversity. Identification of the microbial communities of saline lakes which are some of the most extreme environments on our planet can provide vital information on the evolution of life on Earth, as well as help understand the ecological mechanisms that help maintain the lake’s functional ecosystems [7].

Microorganisms—including bacteria, archaea, and microalgae—form the basis of lake ecosystems [5]. Over the years, sequencing approaches have been used to understand the biogeographical distribution of microbial communities in both freshwater [8,9,10,11,12] and saline lakes [13,14,15,16,17,18]. Within lakes and especially saline lakes, microorganisms are important members with their high genetic diversity playing an essential role in element cycling [19,20]. Salinity has been attributed as one of the major factors (local) influencing the microbial community assembly in saline lake waters [9,21,22,23,24]. Interestingly, the considerable similarity between the biota of freshwater and saline lakes is present between salinity levels of 3 and 20 g·L^−1^. However, with an increase in salinity levels, the microbial community composition of saline lakes diverges from that of freshwater lakes across all taxonomic ranks [25]. Apart from local environmental factors such as salinity, regional factors have also been used to understand the microbial community assembly. Geographical factors have also been known to influence the microbial community assembly with varying scales. Comparison of clone libraries from lakes of Inner Mongolia and Argentina and biogeographical populations of halophilic *Nocardiopsis* and *Streptomyces* isolated from lakes in Central Asia showed geographical variation after more than 700 km [26,27]. Meanwhile, Zorz et al. [17] identified that soda lakes located thousands of kilometers apart have a strikingly similar microbiome. These contrasting results provide scope to compare microbial communities of lakes with varying salinity regimes separated by hundreds of kilometers and located in diverse geographical landscapes to provide detailed insights into the factors governing the microbial community composition.

Mongolia, a landlocked country, is abundant in extremely oligotrophic and saline lakes [28]. Western Mongolia has ~3500 lakes, and more than half are estimated to be saline [28,29]. Western Mongolia has a rich variety of geographical landscapes, including the Gobi Desert and steppe and high mountain environments, and an overall extremely harsh, dry climate with a yearly average precipitation of only ~194 mm. Therefore, lakes in this region have remained relatively untouched from scientific and anthropogenic activity, making them an excellent research environment to explore diversity in high-latitude saline environments and perform a comparative analysis of microbial community compositions. In recent years, anthropogenic activity—especially mining—coupled with global warming has increased the average temperature in the region by 3 to 5 °C. Recent studies have profiled the chemical compositions of 18 saline lakes in western Mongolia [30,31]. However, the microbial community composition of these lakes remains elusive.

In the present study, we examined the bacterial community composition of 18 saline lakes in western Mongolia crisscrossing 7000 km. We sampled across depths of lakes and sequenced 16S rRNA gene amplicons of these samples using paired-end 454 pyrosequencing. This large-scale survey enriches our knowledge of the bacterial community composition of saline lakes situated in Central Asia and attempts to decipher the influence of various salinity regimes and geographical landscapes on the bacterial communities of high-altitude saline lakes. Furthermore, this study will also serve as a vital resource on the microbial community composition of Mongolian lakes, which are experiencing rapid shrinking and are believed to be lost in the future [3].

## 2. Materials and Methods

### 2.1. Study Sites and Sampling

**Figure 1:**
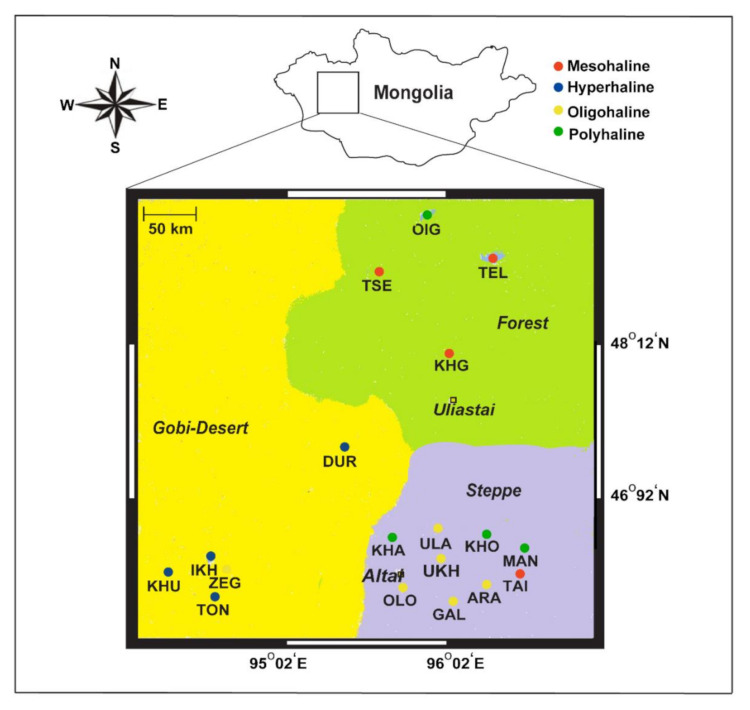
Map showing 18 lakes sampled from western Mongolia, their geographical landscapes (Gobi Desert, forests, and steppe), and their different salinity regimes (hyperhaline, mesohaline, polyhaline, and oligohaline).

We performed sampling in the summer of 2012–2013, the first trip in September 2012 and the second in July 2013, across a large compressional basin bound by the Khangai Mountains, Altai Mountains, a valley of the Great Lakes, and the Gobi Desert in the eastern, western, northern, and southern parts of western Mongolia, respectively. In total, we covered ~7000 km and collected 72 water samples from 18 lakes spanning different salinity regimes (Figure 1). Six-liter lake water samples were vertically collected from each sampling depth at a 1 m interval (at times 0.5 and 0.25 m) using a handmade vacuum deep-water sampler, similar to an earlier study [9]. Collected water samples were retained in 6 L sterile containers and directly transported to the field station (located on the shore of each lake). Water samples were first filtered using a piece of gauze (to remove large debris; 10 µm plankton net), then retentates were filtered with 0.22 µm polycarbonate membrane filters using a vacuum pump at the field station. These membranes were further air-dried at room temperature for 15 min or more and later sealed in separate sterile bags until arrival at the laboratory for DNA extraction.

### 2.2. DNA Extraction, Library Preparation, and Sequencing

Total DNAs were extracted using the cetyltrimethylammonium bromide (CTAB) method. The quality and quantity of extracted nucleic acids were measured using a ScanDrop spectrophotometer (Thermo Scientific, Vantaa, Finland). 16S rRNA gene V6–V8 hypervariable region amplification was performed using the bacterial universal primers 968F (5′-AACGCGAAGAACCTTAC-3′) [32] and 1391R (5′-ACGGGCGGTGWGTRC-3′) [33]

The reaction mixture contained 2.5 U of TaKaRa *Ex taq*^TM^ HS (Takara Bio, Otsu, Japan), 200 µM of each dNTP, 0.2 µM of each primer, and 2–5 ng of diluted template DNA (final concentration: 100 ng/L). PCR was performed under the following conditions: 94 °C for 5 min, 20 cycles at 94 °C for 30 s, 72 °C for 45 s, with a final step at 72 °C for 10 min, and cooling at 4 °C.

The PCR products of the bacterial 16S rRNA gene’s V6–V8 region were verified by DNA agarose gel electrophoresis with a 1% agarose gel with 1X TE buffer and SYBR^®^ Green I. Expectedly sized products (~423 bp) were cut from the gel and purified using a QIAEX II Gel Extraction Kit (Qiagen, Valencia, CA, USA), and quality was verified with a NanoDrop spectrophotometer (Thermo Scientific, Vantaa, Finland).

DNA-tagging PCR was used to tag each PCR product. Seventy-two unique sample-specific four-nucleotide barcodes were added to the 5′ ends of 968F and 1391R primers of each sample. The tagging PCR consisted of an initial step at 94 °C for 5 min, followed by 5 cycles at 94 °C for 30 s, 54 °C for 20 s, and 72 °C for 4 s, with a final step at 72 °C for 10 min, and then cooling at 4 °C. Finally, PCR products were pooled and a 1200 ng mixture of tagged V6–V8 amplicons was subjected to pyrosequencing (Roche GS454 FLX Titanium, Mission Biotech, Taipei, Taiwan).

### 2.3. Measurement and Analysis of Physical and Chemical Parameters

For each lake and depth, both physical and chemical parameters were measured as described previously [30,31]. In brief, temperature, dissolved oxygen, pH, and salinity were measured using a Hanna HI 9828 multiparameter meter (Hanna Instruments, Woonsocket, RI, USA). The pH sensor was calibrated with a 3 M KCl calibration solution (Hanna instruments) each time before measurement. Trace and minor elements in water samples were analyzed by Inductively coupled plasma-mass spectrometry X-Series 2 (Thermo Scientific, Berlin, Germany) in an accredited laboratory (Central Geological Laboratory of Mongolia). Physicochemical parameters used in this study are provided in Supplementary Table S1.

### 2.4. Data Analysis

Reads obtained from paired-end pyrosequencing were quality-filtered with MOTHUR v1.38.1 [34] on a per-sample basis. Quality-filtered reads (minimum length: 360 bp, maximum length: 460 bp, qaverage > 27, homopolymer length < 6) were retained for downstream analysis. Chimeric reads were detected and removed using UCHIME [35] by USEARCH v11 [36], and singletons were also discarded. Qualified sequences were retained for further analysis. Qualified and non-chimeric reads were analyzed with UNOISE3 [37] to obtain zero-radius operational taxonomic units (zOTUs) which are equivalent to amplicon sequence variants. zOTUs were classified with taxonomic labels from the SILVA132 [38,39] database in Mothur on a per-sample basis with a pseudo-bootstrap cutoff of 80%. zOTUs belonging to eukaryote, chloroplast, mitochondria, and unknown were removed before community composition analyses. Bacterial diversity (Shannon and Simpson), richness (Chao1), and evenness (Peilou’s evenness) were calculated using zOTU abundance profiles rarified across all samples. Statistical analysis on α-diversity was performed with the Kruskal–Wallis test, *p* < 0.05.

The relative abundance of zOTUs at the phylum and genus levels was calculated on unrarefied data using an in-house R script (R Team 2018). Bubble plots were generated for the most abundant taxa (genus: >0.01% relative abundance) across all sampling layers of the 18 lakes using ggplot2 [40]. Beta-diversity estimation using non-metric multidimensional scaling (nMDS) analysis was performed after log(x + 1) transforming the zOTU table and calculating Bray–Curtis distance using phyloseq [41] in R. PERMANOVA (using Adonis function) and ANOVA analyses were performed using the vegan package [42] to statistically determine the relationships among bacterial communities and their separation based on salinity type and geographic landscape. Furthermore, zOTUs present in at least 50% of samples with a minimum of 5 reads were used to determine the core bacterial community shared in lakes.

Additionally, functional predictions based on 16S rRNA gene data were performed with the Tax4Fun2 [43] package in R. Tax4Fun2 calculates the metabolic potential of identified microbial taxa by linking taxonomy and abundance profile to a KEGG Orthology (KO) database. Metabolic pathway abundance was normalized by 16S rRNA copy number and used as input to the shotgun data profiling plugin of the web-based tool MicrobiomeAnalyst [44,45]. A linear discriminant analysis (LDA) effect size (LEfSe) analysis was performed to analyze differences in metabolic pathways between different salinity regimes. Statistical significance was tested at the LDA score threshold of >3.5 and *p* < 0.05.

Environmental factors, including physicochemical parameters, were checked for collinearity using Spearman’s rank correlation. Factors with low correlations (*r* < 0.5) were selected for canonical correspondence analysis (CCA) to explore the contributions of these environmental parameters to community structure. Furthermore, ANOVA analysis was performed to assess the significance of constraint variables.

## 3. Results

Water chemistry may help better explain the status of lakes, extrapolate potential interactions between lake water and local geological factors, and assess the impact of anthropogenic activities on aquatic environments. We analyzed the physicochemical profiles of samples from 18 lakes across depths in western Mongolia (Supplementary Table S1). Notably, we categorized the lakes into four types based on salinity: hyperhaline, polyhaline, mesohaline, and oligohaline (Table 1), as outlined by the U.S Fish & Wildlife Service. These lakes were also different in several physicochemical parameters, including temperature, pH, dissolved oxygen, and arsenic concentration. A higher concentration of arsenic was detected in hyperhaline lakes and was particularly high (0.34 mg/L) in Lake Tonkhil (TON). Notably, lakes in forest landscapes were deeper than those in the Gobi Desert and steppe landscapes (Supplementary Table S1).

### 3.1. Bacterial Community Diversity and Richness

**Figure 2:**
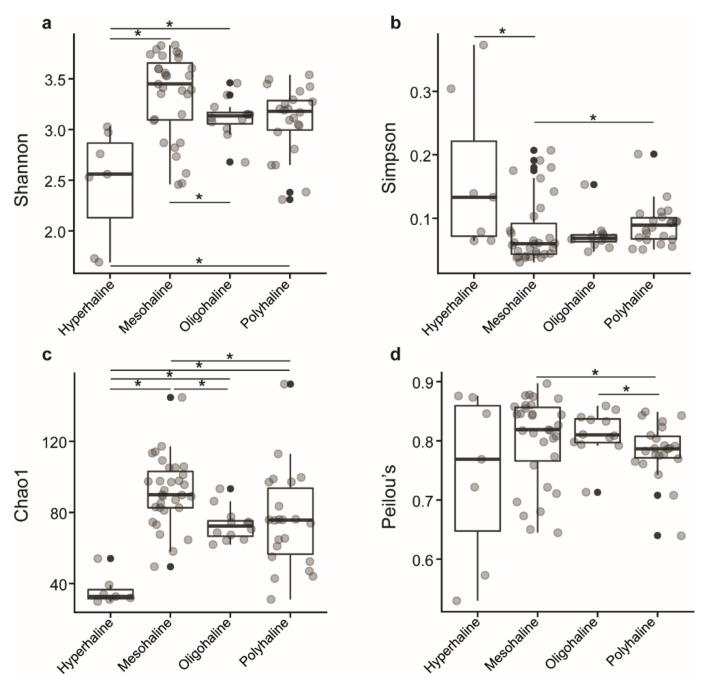
Boxplot of alpha-diversity indices. (**a**) Shannon and (**b**) Simpson indices reflect the diversity of zOTUs in samples. (**c**) Chao1 and (**d**) Peilou’s indices reflect the zOTU abundance and evenness in samples. The greater the Shannon and Simpson indices, the higher the diversity of the microbiota in samples. The greater the Chao1 and Peilou’s indices, the higher the expected species richness and evenness of the microbiota; boxes represent the interquartile range (IQR) between the first and third quartiles (25th and 75th percentiles, respectively), and the horizontal line inside the box defines the median. Whiskers represent the lowest and highest values within 1.5 times the IQR from the first and third quartiles, respectively. “•“ indicates greater than 1.5 times and less than three times the IQR; Grey circles denote respective alpha diversity estimate values of lake samples grouped based on their salinity; “*”: significant difference was tested with the Kruskal–Wallis test, *p* < 0.05.

In total, we observed 663 zOTUs from 182,751 qualified sequences from 72 samples of 18 saline lakes. Of the 663 zOTUs identified, 6 were eukaryotes, 10 were unknown, 3 belong to the chloroplast, and 644 zOTUs were used for alpha diversity analysis. Samples were rarified to an equal depth of 395 reads. Shannon diversity index ranged from 2.46 to 3.83 in mesohaline lakes, 2.69 to 3.46 in oligohaline lakes, 2.31 to 3.49 in polyhaline lakes, and 1.69 to 3.03 in hyperhaline lakes, indicating lower diversity in hyperhaline lakes compared to others. Simpson index ranged from 0.03 to 0.20 in mesohaline, 0.04 to 0.15 in oligohaline, 0.05 to 0.20 in polyhaline, and 0.06 to 0.37 in hyperhaline lakes. Both of these measures were significantly different between lakes of different salinity regimes, Kruskal–Wallis test, *p <* 0.05 (Figure 2a,b).

The Chao1 index, which estimates the number of species that are represented only by a single individual or singletons, and Peilou’s evenness indices, a measure of equality of a community, had a range of 61.9.1–93.3 and 0.71–0.85 in oligohaline lakes, 31.1–152.1 and 0.64–0.84 in polyhaline lakes, 49.5–144.6 and 0.64–0.89 in mesohaline lakes, and 30.0–54.1 and 0.53–0.87 in hyperhaline lakes, respectively. The Chao1 index was significantly different between hyperhaline vs. oligohaline, mesohaline, and polyhaline lakes; oligohaline vs. mesohaline lakes, and mesohaline vs. polyhaline lakes (Kruskal–Wallis test, *p* < 0.05) (Figure 2c), whereas Peilou’s evenness index was significantly different between polyhaline vs. oligohaline and mesohaline lakes (Kruskal–Wallis test, *p* < 0.05) (Figure 2d) only.

### 3.2. Bacterial Community Composition of Western Mongolian Lakes

**Figure 3:**
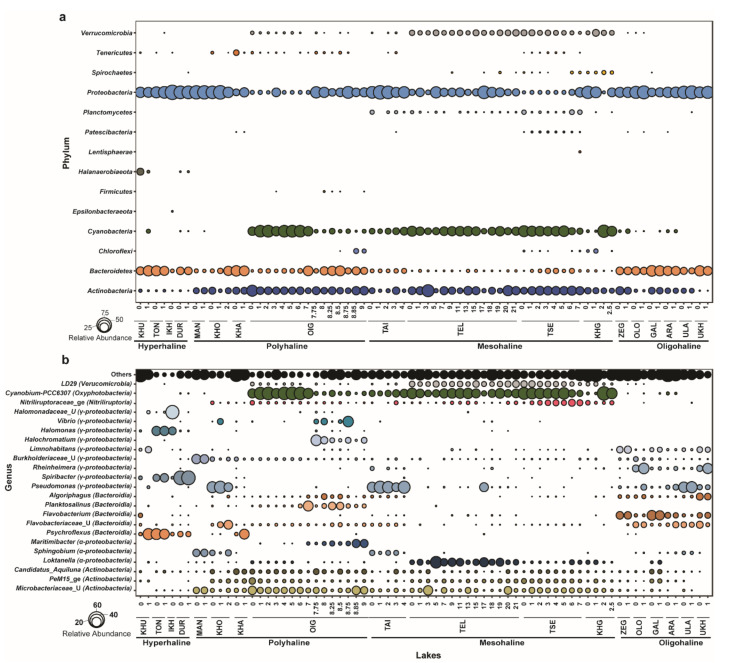
Bacterial community composition of lakes in western Mongolia. (**a**) Relative abundance of bacterial communities at the phylum level (all phyla). (**b**) Relative abundance (>0.01% relative abundance) of bacterial communities at the genus level; taxonomic classes mentioned in brackets.

Out of 644 zOTUs, 18 zOTUs remained unclassified at the phylum level, leaving 626 bacterial zOTUs. These 626 zOTUs were used for downstream analysis. Bacterial community members consisted of 15 phyla and 28 classes, including 4 classes that remained unclassified beyond the phylum. The relative abundance of bacterial communities was determined at each taxonomic level. At the phylum level, *Proteobacteria, Actinobacteria, Bacteroidetes,* and *Cyanobacteria* were dominant. These phyla constituted >90% of the total bacterial community in each lake. *Actinobacteria, Bacteroidetes,* and *Proteobacteria* were identified in all lakes. Phylum *Cyanobacteria* was dominant in polyhaline and mesohaline lakes. *Proteobacteria* was the dominant group in hyperhaline lakes (average (avg.) relative abundance ~63%) and oligohaline lakes (~40% avg. relative abundance), followed by *Bacteroidetes* (hyperhaline: ~30%; oligohaline: ~15%). *Cyanobacteria* were detected mainly in deeper lakes and were particularly dominant in mesohaline lakes across all depths and the oxic zone (0–7 m) of Lake Oigon (OIG), a polyhaline lake. *Actinobacteria* was another major group in mesohaline (~22% avg. relative abundance), polyhaline (~22% average relative abundance), and oligohaline (~18% relative abundance) lakes, but were quite rare in hyperhaline lakes. Many of the dominant phyla were specific to certain lakes. For example, the phylum *Halanaerobiaeota* was only abundant in hyperhaline lakes (~3.5% avg. relative abundance). *Planctomycetes* were dominant only in mesohaline lakes (~1.7% avg. relative abundance) (Figure 3a).

Bacterial genera belonging to classes *Actinobacteria*, *Alpha-* and *Gamma-Proteobacteria*, *Bacteroidia*, *Nitriliruptoria*, *Oxyphotobacteria*, and *Verrucomicrobiae* were the most dominant across all lakes (>0.01% relative abundance). Cyanobium-PCC-6307 (class: *Oxyphotobacteria*) was dominant across all depths in mesohaline lakes (~27.6% avg. relative abundance) and Lake Oigon, whereas *Pseudomonas* (~11% avg. relative abundance) and *Flavobacterium* (~13% avg. relative abundance) were dominant in oligohaline lakes. Polyhaline lakes (except Lake Oigon) harbored unclassified *Microbacteriaceae* as the dominant group. *Psychroflexus* (~21.5% avg. relative abundance), *Spiribacter* (~27% avg. relative abundance), and *Halomonas* (~12% avg. relative abundance) were dominant genera in hyperhaline lakes (Figure 3b).

Furthermore, the shared microbiome among lakes of western Mongolia was deciphered based on the presence of zOTUs in at least 50% of samples with a minimum of five reads, which resulted in only five zOTUs (zOTU12, zOTU23, zOTU64, zOTU88, zOTU556). zOTU12 belongs to the genus *Loktanella* (class: *α-Proteobacteria*) and is one of the dominant zOTUs (avg. relative abundance 3.2%), while zOTU23, zOTU64, zOTU88, and zOTU556 had avg. relative abundances of 2.0%, 1.39%, 1.16%, and 0.35%, respectively, across 72 samples. zOTU23 and zOTU88 belong to *Microbacteriaceae* (unclassified), zOTU64 was annotated to the *Candidatus* Aquiluna genus, and zOTU556 belongs to unclassified *Burkholderiaceae.*

### 3.3. Local, Regional, and Environmental Drivers of Bacterial Community

**Figure 4:**
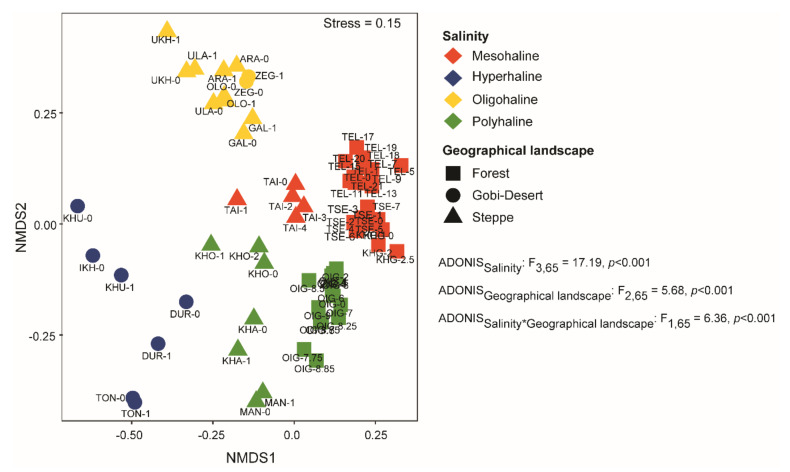
Non-metric multidimensional scaling (nMDS) analysis using Bray–Curtis dissimilarity clustered samples based on salinity and geographical landscape. PERMANOVA analysis was conducted to identify the significant factors shaping the bacterial communities in lakes of western Mongolia.

We used nMDS (based on Bray–Curtis dissimilarity) to compare microbial community compositions among the lakes. At the zOTU level, we observed distinct clusters among lakes based on salinity (Figure 4) and geographical landscape. Both salinity (*p <* 0.001) and geographical landscape (*p <* 0.0001) were significant factors driving community composition in the lakes of western Mongolia.

**Figure 5:**
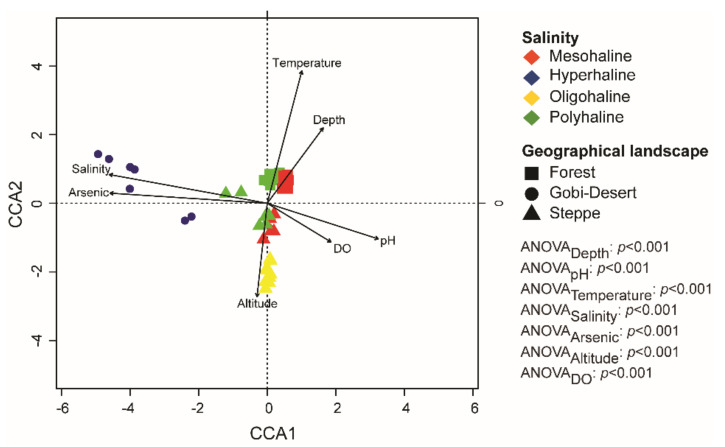
Canonical correspondence analysis (CCA) plot at the genus level. ANOVA analysis was performed to identify the significant environmental factors influencing bacterial communities in lakes of western Mongolia.

Canonical correspondence analysis (CCA) was used to determine the relationships among the environmental variables of each of the lakes, as well as associations between microbial taxa (at the genus level) and physicochemical parameters. Environmental factors (pH, altitude, dissolved oxygen, arsenate, depth, salinity, temperature) were selected based on correlation values >0.5 (Supplementary Figure S1) to perform CCA. Ordination plots show that the bacterial communities were influenced by several environmental factors, such as pH, depth, and salinity (Figure 5). Samples were grouped based on salinity and geographical landscape. Moreover, CCA analysis also indicated that bacterial communities of hyperhaline lakes were significantly associated with arsenic and salinity levels. Furthermore, we observed that lakes in the forest landscape were more tightly clustered than those in the Gobi Desert and steppe landscapes (Figure 5).

### 3.4. Functional Predictions

**Figure 6:**
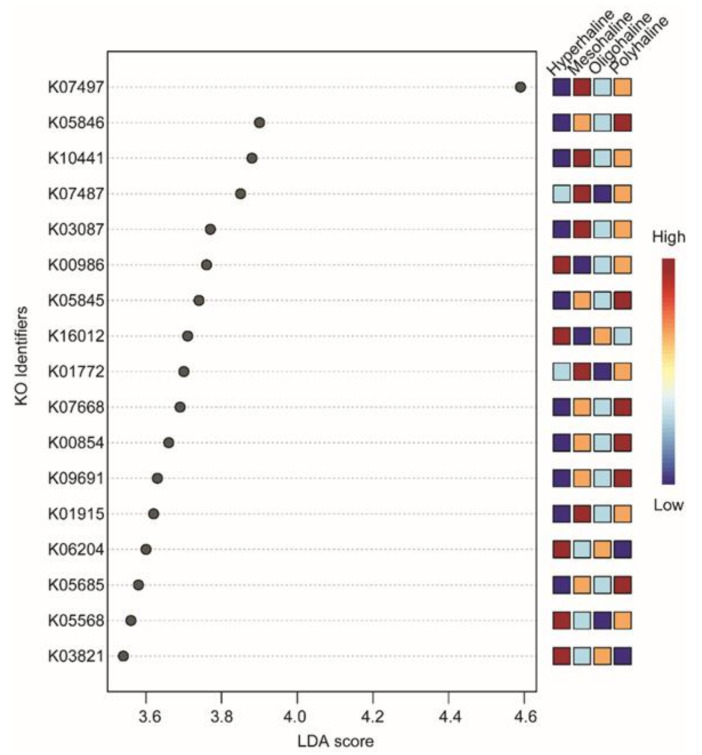
LefSe analysis of predicted functions. LDA score >3.5 and False Discovery Rate (FDR)-corrected *p*-value < 0.05 were considered significant. KO terms were searched in the KEGG database for annotation.

16S rRNA-based functional predictions of the bacterial community identified 6837 KO identifiers. After a low variance filter, 681 KO identifiers were removed, and the remaining 6156 features were scaled (using total sum scaling) before *LEfSe* analysis. The *LEfSe* analysis identified only 17 KO identifiers significantly divergent among different salinity lakes (LDA score threshold of >3.5, FDR-corrected *p* < 0.05). Hyperhaline lakes had five KO identifiers (K00986, K03821, K05568, K06204, K16012) which were significantly divergent. Mesohaline lakes had six KO identifiers which were significantly divergent (K01772, K01915, K0387, K07487, K07497, K10441) and polyhaline lakes also had six significantly divergent KO identifiers (K00854, K05685, K05845, K05846, K09691), and there were no significant identifiers in oligohaline lakes (Figure 6). Most of the significantly divergent KO identifiers belong to ATP-Binding Cassette (ABC) transporters (K05568, K16012, K10441, K05685, K09691, K05845, and K05846) (Table 2).

## 4. Discussion

This study explores the bacterial community composition of 18 remote lakes in western Mongolia with salinity regimes ranging from oligohaline to hyperhaline, situated in arid to semi-arid regions and spanning diverse geographical landscapes. This study is the first to explore the bacterial diversity, composition, and ecology of lakes in western Mongolia. Our findings indicate that both local (water pH, salinity, lake depth) and regional factors (geographical landscape) shape the bacterial community assemblages of these lakes (Figure 4 and Figure 5).

Waters of western Mongolian lakes harbored a wide range of microbial diversity with a Shannon diversity index ranging from 1.69 (DUR-1; hyperhaline) to 3.83 (TAI-3 and TSE-7; mesohaline). Mesohaline lakes had higher microbial diversity (Shannon and inverse Simpson) compared to oligohaline, polyhaline, and hyperhaline lakes (Figure 2a,b). Previous studies on lakes of the Qinghai–Tibetan Plateau, China, and the Monegros Desert, Spain [24,46], have identified comparable microbial diversity with the Shannon index ranging from 3.3 to 6.4 and 1.5 to 2.2, respectively. The microbial richness estimator Chao1 for western Mongolian lakes ranged from 30 (TON-1; Hyperhaline) to 152.1 (OIG-3; Polyhaline) (Figure 2c) and was comparatively lower when compared to Qinghai–Tibetan Plateau lakes which ranged from 1012 to 4499; it is worth noting here that Qinghai–Tibetan Plateau lakes range from freshwater to hyperhaline and the sequencing technology (Illumina vs. 454 pyrosequencing) used among the two studies yield contrasting throughputs. Furthermore, the choice of the hypervariable region selected for amplification, amplicon clustering algorithms, and percentage similarity thresholds also affects the outcomes of community diversity analyses [47]. While the current study amplified the V6–V8 hypervariable region and uses a stringent zOTU approach, Yang et al. [24] amplified V3–V4 and clustered OTUs at 97% similarity. This complicates comparing studies using different approaches and therefore results should be interpreted with caution. In the context of the overall scenario, several studies have found lines of evidence suggesting that microbial diversity decreases with increasing salinity [24,48], and is lowest for hyperhaline lakes, and similar outcomes were observed in our study, reconfirming the previous observations.

Bacterial community composition analysis in the waters of lakes revealed a distinct pattern with the presence of bacterial zOTUs belonging to the phyla *Proteobacteria, Actinobacteria,* and *Bacteroidetes* in all 18 lakes’ water samples. Genus *Cyanobium*-PCC6307 (Phylum: *Cyanobacteria*, Class: *Oxyphotobacteria*) was dominant in mesohaline lakes (Taigan: TAI, Telmen: TEL, Tsegeen: TSE, and Khag: KHG) and in upper water layers (0–7 m) of Lake Oigon (OIG), a polyhaline lake. A total of 79 zOTUs were identified belonging to this genus, members of which are capable of performing photosynthesis and have been identified in both higher-altitude freshwater and saline lakes of Tibet as well as in lakes from other parts of the world [49,50]. Three genera, Ca. Aquilina PeM15 and unclassified *Microbacteriaceae*, and *Nitriliruptoria* of the phylum *Actinobacteria*, which are representatives of aerobic heterotrophs, were the dominant groups in polyhaline, mesohaline, and oligohaline lakes but had lower relative abundance in hyperhaline lakes, which have a salinity range of 51.50–335.5 PSU. *Psychroflexus*, a genus of psychrophilic *Bacteroidetes* initially isolated from Antarctic sea ice and with genomic features providing the capability to thrive not only in low temperature but also in varying salinity, was the dominant phylum in hyperhaline, whereas other *Bacteroidetes* genera *Flavobacterium* and unclassified *Flavobacteriaceae* were dominant in oligohaline lakes and deeper layers (7.75–9 m) of the polyhaline OIG lake (Figure 3a,b). The *psychroflexus* population was also identified to have a recurrent presence and abundance in lakes of Chile and Spain [46,51]. Members of *Bacteroidetes* have been isolated and cultured from both freshwater and marine environments and have been characterized to play an important role in carbon cycling, including metabolizing cellulose, chitin, and other heavy-molecular weight compounds [52], making them cosmopolitan bacteria in aquatic environments. Other dominant genera were from classes α and γ-*Proteobacteria*. *Limnohabitans* a well-known planktonic bacteria genus of freshwater [19], was dominant in oligohaline lakes, which had the lowest salinity range (0.33–2.27 PSU) in studied lakes. Genera *Halomonas* and *Spiribacter* were dominant in hyperhaline lakes, and members of these genera have been classified as moderate halophiles, identified and isolated from environments with moderate to high salinity such as saltern ponds and marine environments [53]. Interestingly, *Spiribacter* species lack chemolithotrophic capabilities, and no carbon fixation pathways have been detected in the sequenced genomes. Furthermore, the LD29 lineage from phylum *Verrucomicrobia* had higher relative abundances in TEL and TSE, but low relative abundance in Taigan (TAI) and Khag (KHG) lakes, even though they are mesohaline (Figure 2b). The LD29 lineage and other members of *Verrucomicrobia* were recently reported to be abundant in a Baltic Sea salinity transect and other brackish water environments [54,55,56]. Members of *Verrucomicrobia* are cosmopolitan in origin and found in various aquatic bodies, including rivers and freshwater lakes [11,12,19]. Furthermore, members of *Verrucomicrobia* can metabolize plankton-derived organic matter [57]. This relationship was observed in polyhaline and mesohaline lakes in the current study, which are dominated by pico-cyanobacteria and *Verrucomicrobia*.

Identification of a highly reduced shared microbiome with only five zOTUs with average relative abundance >0.1% among all the lakes belonging to the dominant phyla *Proteobacteria* and *Actinobacteria* showcases the cosmopolitan nature of members of these phyla but also points to the tremendous diversity shaped putatively by multiple factors. All these bacterial phyla have been known to harbor bacterial species capable of thriving in various saline environments and contribute a significant proportion of the microbial community in earlier studies as well [23,24,48,58,59,60,61].

As microbial communities can be influenced by environmental conditions, variations in the environment both at the local and regional levels could be correlated with microbial community assembly. Since the lakes in our study had diverse physicochemical parameters (Supplementary Table S1) and were also situated in very different geographical landscapes (steppe, Gobi Desert, and forests), it is important to delineate the factors influencing the community composition. Beta-diversity analysis (based on Bray–Curtis dissimilarity) at the zOTU level appeared to cluster lakes based on salinity regimes and geographical landscapes (Figure 3). Furthermore, PERMANOVA (Adonis) analysis suggested that the bacterial community compositions of lakes are governed by salinity (ADONIS_salinity_: F_3,65_ = 17.91, *p <* 0.001), geographical landscape (ADONIS_Geographical Landscape_: F_2,65_ = 5.68, *p <* 0.001), and also a combination of the two (ADONIS_salnity*Geograhical Landscape_: F_1,65_ = 6.36, *p <* 0.01). In recent studies, salinity has been proposed to be the major factor driving community dissimilarity among lakes [9,21,23,24], and none have reported the influence of geographical landscape on the community assembly.

CCA analysis at the genus level yielded interesting patterns, as bacterial communities in lakes were significantly related to both local and regional factors (Figure 4). The hypersaline lakes—all from the Gobi Desert—were positively correlated with salinity and arsenic concentration, whereas the mesohaline and polyhaline lakes spanning the forest landscape clustered strongly together and were significantly positively correlated with temperature and depth (Figure 4). Furthermore, all of the lakes in the steppes, irrespective of their salinity regime (mesohaline, polyhaline, and oligohaline), were positively correlated with altitude (Figure 4). Our results highlight the importance of the combination of local and regional factors in influencing bacterial community structures in lakes. Furthermore, the impact of regional factors like geographical distance on community composition and diversity has been reported to be scale-dependent [62]. Local environmental factors are reported to have a higher influence over small spatial scales, whereas regional factors play a significant role when dealing with large distances of tens of thousands of kilometers [17,62]. Therefore, our study’s clustering of lakes in the same landscape corroborates the scale-dependent influence of regional factors. Further, the influence of local factors in shaping the bacterial community is evident through our results.

The ubiquitous presence of cosmopolitan bacteria at a higher taxonomic rank (phyla *Proteobacteria*, *Actinobacteria,* and *Bacteroidetes*) has been reported in lake waters from lakes in the Qinghan–Tibetan Plateau, Mongolia [24], as well as other regions of the world [17,46]. A comprehensive analysis at the lower taxonomic rank (genus) on western Mongolian lakes suggested habitat specialization of specific taxonomic groups, e.g., *Spiribacter, Cyanobium,* and *Halomonas* (Figure 2a,b). Functional prediction using 16S rRNA gene abundance as a proxy to the metabolic profile identified differences in functional traits of lakes from different salinity regimes. Though all the lakes had KO identifiers from ATP-Binding Cassette Transporters as the most significantly different terms, hyperhaline lakes also had K03821 (butanoate metabolism) and K06204 (biofilm formation) as the significantly different KO identifiers compared to other lakes. Interestingly, none of the KO identifiers were significantly different in oligohaline lakes when compared with other salinity regimes lakes (Figure 6). Identification of different ABC transporters including the osmoprotectant ABC transport system (K05845 and K05846), multicomponent Na^+^-H^+^ antiporter system (K05568), and carbohydrate metabolism identifiers (K01915, K03821, K00854) shows finer scale functional trait variability of microbes in these lakes. Although it is difficult to identify which bacteria species or groups carry out most of these functions, this observation provides preliminary insights into the metabolic functioning of these lakes which should be further explored using a functional metagenomics approach.

## 5. Conclusions

Our study describes, for the first time, the bacterial community composition diversity, richness, and ecological aspects of remote lakes of western Mongolia. High bacterial diversity in meso-, poly-, and oligohaline lakes points out salinity as a major driver for community diversity and the role of bacteria in biogeochemical processes in these lakes. We also identified that both local and regional factors influence the community composition of lakes. These results provide basic information about the very few undisturbed aquatic environments that could help guide future investigations into the long-term environmental impacts on these pristine ecosystems of landlocked Mongolia.

## Figures and Tables

**Table 1:** Location of sampling sites, their altitude, landscape, and abbreviated codes.

Salinity	Lake Name (Trip)	Code	Latitude N	Longitude E	Altitude (m)	Landscape
Hyperhaline	Khulma (1)	KHU	46°11′01	93°32′39	2224	Gobi Desert
	Tonkhil (1)	TON	46°11′08	93°54′16	2063	Gobi Desert
	Ikhes (1)	IKH	46°27′39	94°03′54	1601	Gobi Desert
	Duruu (1)	DUR	47°16′45	95°42′35	1425	Gobi Desert
Polyhaline	Mangas (1)	MAN	46°29′52	96°47′47	1760	Steppe
	Kholboo (1)	KHO	46°24′02	97°18′51	1799	Steppe
	Khadaasan (1)	KHA	46°30′25	96°17′16	2003	Steppe
	Oigon (1)	OIG	49°09′03	96°31′36	1668	Forest
Mesohaline	Taigam (1)	TAI	46°22′09	97°24′06	1790	Steppe
	Telmen (2)	TEL	48°51′37	97°19′34	1795	Forest
	Tsegeen (2)	TSE	48°44′00	95°51′32	1875	Forest
	Khag (2)	KHG	48°04′29	96°38′28	2036	Forest
Oligohaline	Zegst (1)	ZEG	46°12′18	93°55′35	2066	Gobi-Desert
	Olon (1)	OLO	46°18′49	96°22′13	2185	Steppe
	Galuut (2)	GAL	46°18′03	96°42′23	2034	Steppe
	Argashuun (1)	ARA	46°28′54	97°08′01	1798	Steppe
	Ulaan (1)	ULA	46°27′20	96°17′08	1996	Steppe
	Ulaan- Kholboo (1)	UKH	46°27′10	96°17′01	1997	Steppe

Altitude (m)—above sea level.

**Table 2:** Differentially abundant KO identifiers in different salinity regimes, their KEGG annotation, and pathways.

KO Identifier	KEGG Orthology	KEGG Pathway/BRITE	Salinity Regime
K00986	RNA-directed DNA polymerase	Unclassified *	Hyperhaline
K16012	ATP-binding Cassette, CydC	ABC transporters	Hyperhaline
K06204	DnaK suppressor protein	Biofilm formation	Hyperhaline
K05568	Multicomponent Na^+^: H^+^ antiporter	Transporters	Hyperhaline
K03821	Polyhydroxyalkanoate-synthase subunit PhaC	Butanoate metabolism	Hyperhaline
K07497	Putative transposase	Unclassified	Mesohaline
K10441	Ribose transport system ATP-binding protein	ABC transporter	Mesohaline
K07487	Transposase	Unclassified	Mesohaline
K03087	RNA polymerase nonessential primary-like sigma factor protein	Biofilm formation	Mesohaline
K01772	Protoporphyrin/coproporphyrin ferrochelatase	Porphyrin and chlorophyll metabolism	Mesohaline
K01915	Glutamine synthetase	Carbohydrate metabolism	Mesohaline
K05845	Osmoprotectant transport system substrate-binding protein	ABC transporters	Polyhaline
K05846	Osmoprotectatnt transport system substrate-binding protein	ABC transporters	Polyhaline
K07668	Two-component system, OmpR family, response regulator VicR	Two-component system	Polyhaline
K00854	Xylulokinase	Pentose and glucuronate interconversions	Polyhaline
K09691	Lipopolysaccharide transport system ATP-binding/permease protein	ABC transporters	Polyhaline
K05685	Macrolide transport system ATP-binding/permease protein	ABC transporters	Polyhaline

* Unclassified: not classified in “Pathways” or BRITE in the KEGG database.

## Supplementary Materials

The following are available online at https://www.mdpi.com/2076-2607/8/11/1729/s1, Figure S1: Correlation values of the parameters used to correlate microbial community composition with environmental parameters using canonical correspondence analysis. Table S1: Physicochemical parameter profile of western Mongolian lakes.
