# Supplementary material for: A Large-Scale Survey of the Bacterial Communities in Lakes of Western Mongolia with Varying Salinity Regimes
Source: Microorganisms. 2020 Nov 4;8(11):1729. doi: 10.3390/microorganisms8111729 (PMC7716208; doi:10.3390/microorganisms8111729)
Supplement: Supplementary file 1 [file microorganisms-08-01729-s001.zip › microorganisms-987716-supplementary.docx]

A Large-Scale Survey of the Bacterial Communities in Lakes of Western Mongolia with Varying Salinity Regimes

Kshitij Tandon ^1,2,3,†^, Bayanmunkh Baatar ^1,4,†^, Pei-Wen Chiang ^1^, Narangarvuu Dashdondog ^4^, Bolormaa Oyuntsetseg ^4,^* and Sen-Lin Tang ^1,2,^*

^1^ Biodiversity Research Center, Academia Sinica, Taipei 115, Taiwan; kshitijtandon@gate.sinica.edu.tw (K.T.); b.bayanmunkh@mnun.edu.mn (B.B.); momo12390@gmail.com (P.-W.C.)

^2^ Bioinformatics Program, Institute of Information Science, Taiwan International Graduate Program, Academia Sinica, Taipei 115, Taiwan

^3^ Institute of Molecular and Cellular Biology, National Tsing Hua University, Hsinchu 300, Taiwan

^4^ School of Arts and Sciences, National University of Mongolia, Ulaanbaatar 14201, Mongolia; garvuu@num.edu.mn

***** Correspondence: bolormaa@num.edu.mn (B.O.); sltang@gate.sinica.edu.tw (S.-L.T.)

**^†^** These authors contributed equally to this work.

**Supplementary Figures and Tables**


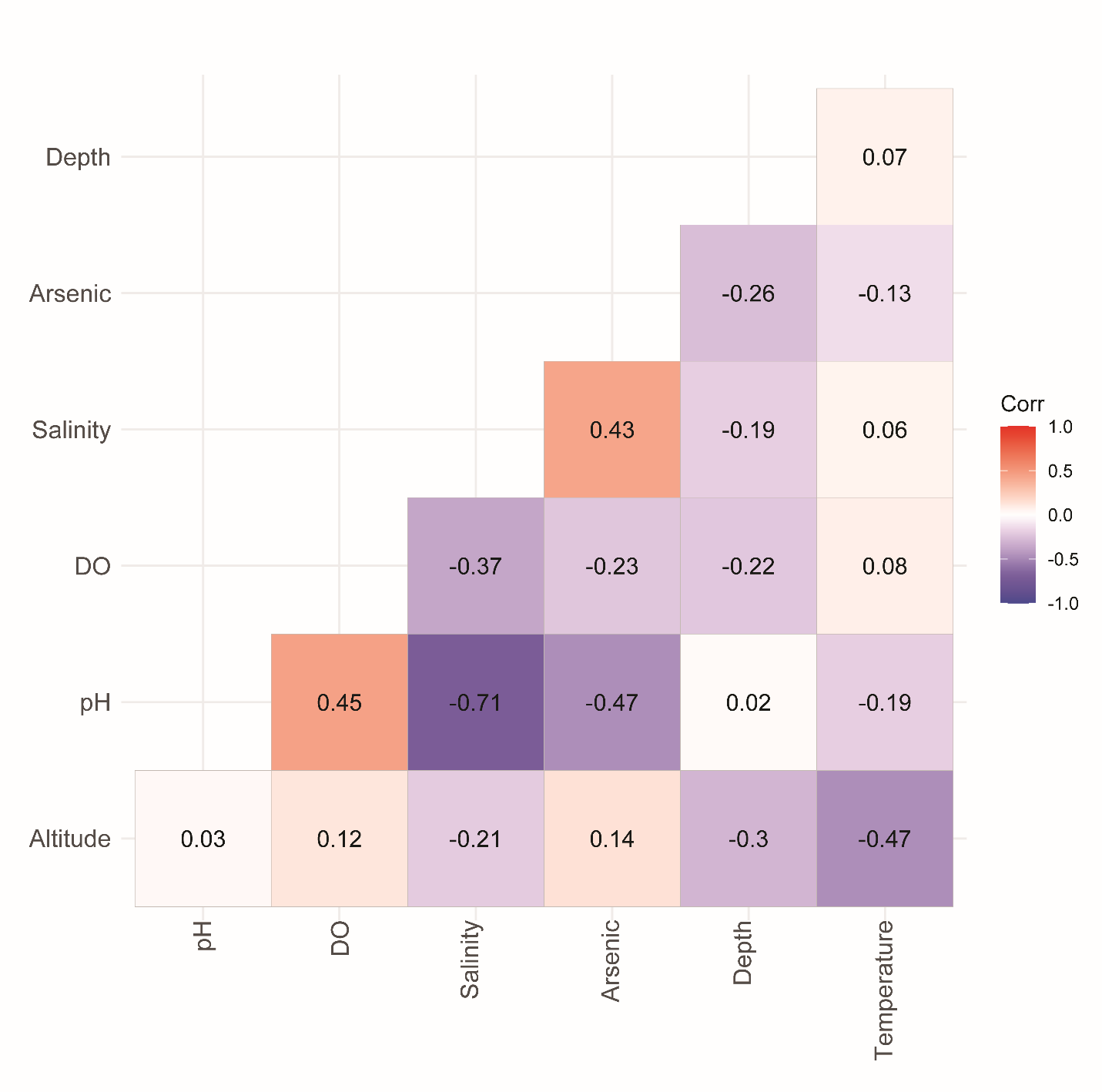


**Supplementary Figure S1.** Correlation values of the parameters used to correlate microbial community composition with environmental parameters using Canonical Correspondence Analysis.

**Table S1.** Physicochemical parameter profile of western Mongolian lakes

| **Salinity** | **Lake code** | **Depth(m)** | **T (°C)** | **pH** | **Salinity (PSU^a^)** | **DO (mg/l)** | **As (mg/l)** |
| --- | --- | --- | --- | --- | --- | --- | --- |
| Hyperhaline | KHU | 0 | 13.21 | 7.1 | 234.96 | 0.13 | 0.07 |
|  |  | 1 | 13.21 | 7.1 | 234.96 | 0.13 | 0.06 |
|  | TON | 0 | 10.66 | 8.31 | 51.50 | 0.6 | 0.23 |
|  |  | 1 | 13.18 | 8.06 | 62.69 | 0.65 | 0.34 |
|  | IKH | 0 | 10.63 | 8.03 | 137.42 | 0.6 | 0.21 |
|  | DUR | 0 | 13.15 | 7.98 | 335.50 | 1.79 | 0.10 |
|  |  | 1 | 16.17 | 8.22 | 335.50 | 1.35 | 0.09 |
| Polyhaline | MAN | 0 | 12.77 | 9.25 | 22.47 | 3.31 | N.D |
|  |  | 1 | 12.67 | 9.26 | 22.46 | 3.19 | N.D |
|  | KHO | 0 | 12.19 | 9.62 | 16.94 | 5.69 | 0.06 |
|  |  | 1 | 12.24 | 8.99 | 16.93 | 5.54 | 0.06 |
|  |  | 2 | 12.36 | 9.04 | 16.94 | 6.04 | 0.07 |
|  | KHA | 0 | 11.03 | 10.13 | 30.49 | 3.27 | 0.06 |
|  |  | 1 | 10.99 | 9.83 | 30.64 | 2.98 | 0.08 |
|  |  | 0 | 18.57 | 9.02 | 24.76 | 6.53 | N.D |
|  |  | 1 | 19.41 | 9.25 | 24.56 | 4.98 | N.D |
|  |  | 2 | 19.32 | 9.23 | 24.54 | 4.75 | N.D |
|  |  | 3 | 20.39 | 8.96 | 24.11 | 3.71 | N.D |
|  |  | 4 | 20.81 | 8.95 | 24.09 | 2.95 | N.D |
|  |  | 5 | 19.49 | 8.99 | 24.55 | 3.81 | N.D |
|  |  | 6 | 18.30 | 9.02 | 24.73 | 2.76 | N.D |
|  | OIG | 7 | 14.27 | 9.04 | 28.42 | 1.55 | N.D |
|  |  | 7.75 | 15.23 | 8.84 | 34.28 | 0.36 | N.D |
|  |  | 8 | 12.28 | 9.10 | 29.09 | N.D | N.D |
|  |  | 8.25 | 14.61 | 8.88 | 34.09 | N.D | N.D |
|  |  | 8.5 | 13.99 | 8.90 | 33.76 | N.D | N.D |
|  |  | 8.75 | 14.42 | 8.97 | 31.98 | N.D | N.D |
|  |  | 8.85 | 14.40 | 8.92 | 32.08 | N.D | N.D |
|  |  | 9 | 14.52 | 8.88 | 28.05 | N.D | N.D |

| **Salinity** | **Lake code** | **Depth(m)** | **T (°C)** | **pH** | **Salinity (PSU^a^)** | **DO (mg/l)** | **As (mg/l)** |
| --- | --- | --- | --- | --- | --- | --- | --- |
| Mesohaline | TAI | 0 | 11.9 | 9.64 | 9.19 | 4.11 | N.D |
|  |  | 1 | 11.83 | 9.09 | 9.17 | 4.14 | N.D |
|  |  | 2 | 12.16 | 8.96 | 9.25 | 4.09 | N.D |
|  |  | 3 | 12.03 | 9.00 | 9.25 | 3.49 | N.D |
|  |  | 4 | 11.56 | 9.13 | 9.34 | 0.15 | N.D |
|  | TEL | 0 | 16.44 | 9.02 | 5.30 | 7.11 | N.D |
|  |  | 1 | 18.76 | 9.08 | 1.40 | 6.93 | N.D |
|  |  | 3 | 18.92 | 9.19 | 1.40 | 6.48 | N.D |
|  |  | 5 | 19.42 | 9.10 | 5.20 | 6.33 | N.D |
|  |  | 7 | 15.92 | 9.19 | 5.20 | 6.59 | N.D |
|  |  | 9 | 15.27 | 9.22 | 5.20 | 6.36 | N.D |
|  |  | 11 | 14.07 | 9.56 | 5.20 | 6.19 | N.D |
|  |  | 13 | 12.18 | 9.22 | 5.30 | 6.00 | N.D |
|  |  | 15 | 12.61 | 9.06 | 5.30 | 4.63 | N.D |
|  |  | 17 | 11.95 | 9.09 | 5.40 | 4.26 | N.D |
|  |  | 18 | 10.47 | 9.28 | 5.50 | 2.94 | N.D |
|  |  | 19 | 10.07 | 9.02 | 5.50 | 2.50 | N.D |
|  |  | 20 | 15.53 | 8.96 | 4.90 | 1.39 | N.D |
|  |  | 21 | 12.27 | 8.99 | 5.40 | 1.37 | N.D |
|  | TSE | 0 | 17.31 | 9.84 | 9.97 | 6.27 | N.D |
|  |  | 1 | 16.92 | 9.21 | 9.91 | 4.34 | N.D |
|  |  | 2 | 17.03 | 9.21 | 9.95 | 4.12 | 0.02 |
|  |  | 3 | 16.59 | 9.07 | 9.90 | 3.63 | 0.02 |
|  |  | 4 | 16.62 | 8.99 | 10.02 | 3.04 | 0.02 |
|  |  | 5 | 16.18 | 9.06 | 9.99 | 2.17 | 0.02 |
|  |  | 6 | 15.61 | 8.95 | 10.00 | 2.71 | 0.02 |
|  |  | 7 | 15.55 | 8.76 | 9.97 | 1.57 | N.D |
|  | KHG | 0 | 18.12 | 9.06 | 8.18 | 5.55 | 0.11 |
|  |  | 1 | 16.17 | 8.87 | 8.22 | 5.62 | 0.11 |
|  |  | 2 | 15.48 | 8.7 | 8.17 | 0.43 | 0.11 |
|  |  | 2.5 | 15.13 | 8.98 | 8.46 | 3.09 | 0.10 |
| Oligohaline | ZEG | 0 | 10.97 | 9.12 | 0.72 | 4.03 | N.D |
|  |  | 1 | 10.97 | 9.12 | 0.72 | 10.9 | N.D |
|  | OLO | 0 | 3.62 | 9.68 | 0.33 | 1.67 | N.D |
|  |  | 1 | 3.57 | 9.53 | 0.33 | 1.68 | N.D |
|  | GAL | 0 | 7.22 | 9.4 | 1.27 | 2.66 | N.D |
|  |  | 1 | 6.61 | 9.39 | 1.27 | 2.63 | N.D |
|  | ARA | 0 | 13.38 | 8.94 | 0.59 | 4.89 | N.D |
|  |  | 1 | 10.47 | 8.74 | 0.6 | 4.82 | N.D |
|  | ULA | 0 | 6.6 | 9.35 | 2.27 | 6.08 | N.D |
|  |  | 1 | 6.16 | 9.73 | 2.26 | 5.79 | N.D |
|  | UKH | 0 | 6.09 | 9.16 | 1.98 | 6.26 | N.D |
|  |  | 1 | 6.16 | 9 | 1.96 | 6.1 | N.D |

Depth (m)-0 –surface water, ^a^PSU-Practical Salinity Unit
